# Novel Superabsorbent Hydrogels Based on Polyacrylamide and White Angico Gum Enhanced with Kaolinitic Clay and Soapstone for Potential Agricultural Applications

**DOI:** 10.3390/ijms27094150

**Published:** 2026-05-06

**Authors:** Angelina Santos de Carvalho, Arthur Francisco de Paiva Alcântara, Vicente de Sousa Marques, Ariane Maria da Silva Santos, Ronaldo Cunha Coelho, Edvani Curti Muniz

**Affiliations:** 1 Graduate Program in Materials Science and Engineering, Federal University of Piauí, Teresina 64049-550, Brazil; angelinasantos653@gmail.com; 2 Teaching Department, Federal Institute of Piauí (IFPI), Valença Campus, Valença 64300-000, Brazil; arthur.alcantara@ifpi.edu.br; 3 Teaching Department, Federal Institute of Maranhão (IFMA), Codó Campus, Maranhão 65400-000, Brazil; vsmarques7@gmail.com; 4 Department of Chemistry, Federal University of Piauí, Teresina 64049-550, Brazil; ariane.am42@gmail.com; 5 Teaching Department, Federal Institute of Piauí (IFPI), Campus Teresina Central, Teresina 64000-040, Brazil; ronald@ifpi.edu.br; 6 Graduate Program in Chemistry, State University of Maringá, Maringá 87020-900, Brazil

**Keywords:** superabsorbent hydrogels, polyacrylamide, white angico, kaolinitic clay, soapstone, agriculture

## Abstract

Population growth and climate change demand technologies for the efficient use of water in agriculture. This study aimed to synthesize and characterize hybrid hydrogels of polyacrylamide and white angico gum (*Anadenanthera colubrina*), reinforced with kaolinitic clay and soapstone, for potential application as soil conditioners and nutrient carriers. The hydrogels were obtained via radical polymerization, followed by alkaline hydrolysis (0.1 mol L^−1^ NaOH) to convert amide groups into carboxylates. The results indicated that the HPAD formulation [constituted by white angico gum (1:1); 5% (w/w) kaolin and 5% (w/w) steatite (soapstone)] presented the best balance, with a maximum compressive force greater than 200 N, thermal stability up to 310 °C, and a swelling capacity of 60 g/g in saline medium, surpassing the limits of viability for use in soil. The kinetics followed the pseudo-second-order model, and the point of zero charge (pH 9.0–11.7) favored phosphate retention. It is concluded that the HPAD hydrogel, one of several hydrogel formulations developed in this study, is a viable and safe technical alternative, with non-toxicity exceeding 80% in *Artemia salina* assays and capable of optimizing water and nutrient efficiency in agricultural systems.

## 1. Introduction

Population growth and climate change impose on agriculture the challenge of increasing productivity sustainably in the face of water scarcity and soil degradation [1,2]. In this context, superabsorbent hydrogels emerge as a strategic technology capable of retaining large volumes of water and releasing them gradually, minimizing losses due to leaching [3,4].

The development of synthetically derived superabsorbent hydrogels based on acrylic acid and acrylamide has been widely reported due to their straightforward synthesis and high water absorption capacity at a relatively low cost [5]. However, these synthetic materials are not environmentally friendly and are not biodegradable [2]. In this context, the use of natural polymers such as polysaccharides has been explored to impart biodegradability and low toxicity to hydrogels [6]. In Brazil, polysaccharides such as cashew gum, babassu mesocarp, and gum Arabic have been widely investigated for hydrogel synthesis [1,6,7]. White angico gum is a polysaccharide that has recently gained increasing attention in the literature and is found in several regions of Brazil. White angico (AB) shows potential for application in superabsorbent hydrogels due to its hydrophilic functional groups and structural versatility [6,8]. In addition, reinforcing fillers such as kaolinitic clay (AC) are commonly used to improve the mechanical properties of hydrogels and have attracted considerable interest in the formulation of polymeric materials [9]. Kaolinite is widely available in several regions of Brazil [9]. Steatite (PS), a regional metamorphic rock, has also attracted attention due to its interesting properties, including the ability to release nutrients in addition to providing mechanical reinforcement [10,11]. Although still little explored in agricultural applications, steatite appears to be a promising material for such purposes.

White angico gum is a polysaccharide obtained from the stem exudate of the *Anadenanthera colubrina* tree, a species native to South America and widely found in Brazil. It produces a highly viscous and biodegradable natural gum. This polysaccharide is classified as an arabinogalactan, a heteropolysaccharide composed of approximately 63–67% arabinose, 20% galactose, 10% glucuronic acid, and 6% rhamnose [12]. The main chain of white angico gum consists of β-D-galactopyranose (1→3) units, with side chains of varying composition, including oligosaccharides containing α-arabinofuranose, α-rhamnopyranose, and β-glucopyranosyl acid units [13].

In addition to the polysaccharide component, the hydrogel system developed in this study incorporates mineral materials as reinforcing agents and structural modifiers. In this context, kaolinitic clay, commercially known as kaolin, is mainly composed of the mineral kaolinite [9], a hydrated aluminosilicate with the chemical formula Al_2_Si_2_O_5_(OH)_4_. Its composition is approximately 46% SiO_2_, 40% Al_2_O_3_, and 14% H_2_O [9]. The structure of kaolinite consists of stacked 1:1 layers formed by a tetrahedral silica sheet and an octahedral alumina sheet [14,15]. Additionally, steatite, used as a complementary mineral filler, is primarily composed of talc, with the chemical formula Mg_3_Si_4_O_10_(OH)_2_, and has an approximate composition of 31.7% MgO, 63.5% SiO_2_, and 4.8% H_2_O [11,16].

The objective of this work is to synthesize and characterize hybrid hydrogels based on polyacrylamide and white angico gum, reinforced with abundant Brazilian minerals such as kaolinitic clay and steatite, aiming at their application as soil conditioners and systems for phosphate adsorption and controlled release. It is hypothesized that the incorporation of white angico gum (AB) introduces hydrophilic functional groups into the hydrogel network while providing a renewable, non-toxic, biodegradable, and low-cost component. Such polysaccharide-based hydrogels are expected to enhance water retention capacity and improve soil porosity, thereby favoring plant growth and agricultural productivity [3,17]. Furthermore, mineral fillers such as kaolinitic clay (AC) are expected to act as reinforcing agents, contributing to improved mechanical stability and providing additional hydrophilic sites [9,18]. Steatite (PS) is also expected to enhance mechanical resistance while potentially supplying nutrients beneficial for plant development [10,19]. These incorporations contribute to the development of more resilient and efficient materials for potential agricultural applications. The combination of polyacrylamide copolymerization with these natural (mineral and non-mineral) components is expected to produce a synergistic effect, resulting in hydrogels with improved mechanical strength, water retention capacity, and nutrient interaction potential, making them suitable for agricultural applications. Finally, to clarify the experimental design, seven hydrogel formulations were synthesized and grouped as follows. Group 1 consists of polysaccharide-containing hydrogels: HPAB, HPAC, HPPS, and HPAD. Group 2 consists of polysaccharide-free hydrogels: HSAC, HSPS, and HSAD. All formulations contain acrylamide as a common monomer, ensuring a consistent polymeric backbone for comparison.

## 2. Results

### 2.1. Fourier Transform Infrared (FTIR) Analysis

**Figure 1:**
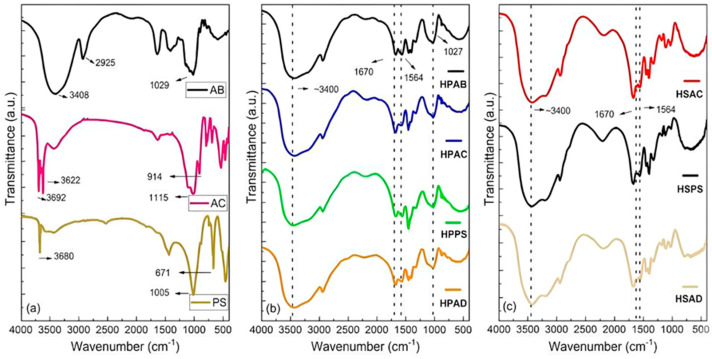
FTIR spectra of (**a**) raw materials (white angico gum (AB), kaolinitic clay (AC), and soapstone (PS)); (**b**) Group 1 hydrogels (polyacrylamide-based systems containing purified white angico gum: HPAB, HPAC, HPPS, and HPAD); and (**c**) Group 2 hydrogels (polyacrylamide–mineral systems without gum: HSAC, HSPS, and HSAD).

The molecular structures and chemical interactions in the synthesized hydrogels were analyzed by FTIR, as shown in Figure 1. A comparative analysis of the starting materials (Figure 1a) and the composites (Figure 1b,c) allowed for the identification of polymer network formation and the incorporation of mineral fillers.

According to Figure 1, the FTIR spectrum of white angico gum (AB) exhibited bands at 3400 cm^−1^ (O–H stretching) and 2925 cm^−1^ (aliphatic C–H stretching). In the fingerprint region, the bands at 1076 and 1029 cm^−1^ are attributed to C–O–C glycosidic bonds and the angular deformation of O–H groups [20].

For the minerals, the spectrum of kaolinitic clay (AC) showed bands at 3692 and 3650 cm^−1^, attributed to external hydroxyls, and at 914 cm^−1^, attributed to internal octahedral hydroxyls [9,21,22]. Si–O–Si vibrations were identified at 1115 and 1012 cm^−1^. Steatite (PS) exhibited characteristic peaks at 3680–3674 cm^−1^ and 671 cm^−1^ [16,23]. In the synthesized hydrogels, the bands at 1368 and 1029 cm^−1^ were retained [24,25]. The bands at 1670 cm^−1^ (C=O stretching, Amide I) and 1564 cm^−1^ (asymmetric COO^−^ stretching) were maintained in all hydrogel FTIR spectra [9]. A broadening of the band at 3400 cm^−1^ was observed in all hydrogel FTIR spectra. The incorporation of mineral fillers was evidenced by a reduction in the intensity of the hydroxyl bands (3400 cm^−1^) and by the shift in the Si–O and Al–OH peaks.

### 2.2. X-Ray Diffraction Analysis

**Figure 2:**
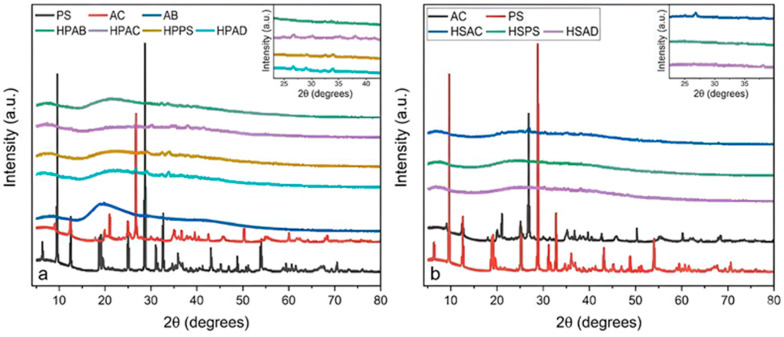
XRD diffractograms of (**a**) Group 1 hydrogels (polyacrylamide–gum-based systems) together with their precursors; and (**b**) Group 2 hydrogels (polyacrylamide–mineral systems) together with their precursors.

The diffraction patterns of the precursors and the synthesized hydrogels were evaluated by X-ray diffraction (XRD), as shown in Figure 2.

The diffractogram of white angico gum (AB) exhibited a broad diffraction halo, with a characteristic maximum centered at 2θ = 19.5° [20]. For the pure mineral materials (Figure 2a), kaolinitic clay (AC) showed well-defined crystalline peaks at 2θ = 12.3°, 20°, and 25°, along with quartz reflections at 2θ = 22° [9]. Steatite (PS) exhibited a high degree of crystallinity, with the characteristic peak of talc (its main constituent) at 2θ = 28.8° [16].

In all synthesized hydrogels (Figure 2a,b), an amorphous phase predominates, as evidenced by the halo between 2θ = 15° and 30°. In hydrogels containing the polysaccharide, a reduction in the intensity of the halo characteristic of the pure gum was observed [20]. For composites containing mineral fillers, the characteristic AC and PS peaks were diminished or disappeared after incorporation into the network.

### 2.3. Swelling in Distilled Water

**Figure 3:**
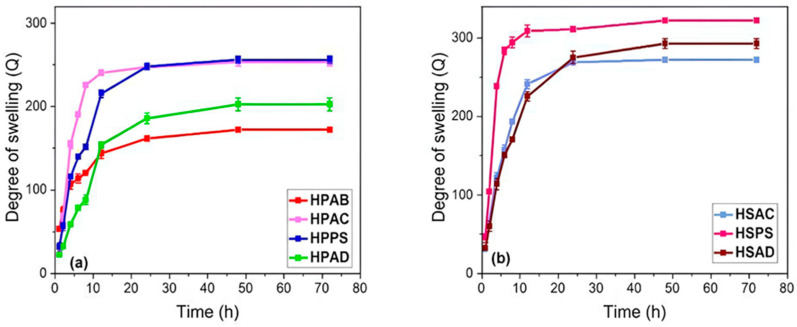
Swelling degree (Q) of the synthesized hydrogels in distilled water: (**a**) Group 1 (gum-based systems: HPAB, HPAC, HPPS, and HPAD); and (**b**) Group 2 (gum-free systems: HSAC, HSPS, and HSAD).

According to Figure 3, the formulations reached swelling equilibrium after approximately 30–35 h. The hydrogels from Group 1 (containing white angico gum) showed a lower swelling capacity compared to those from Group 2 (systems without gum). Among the formulations tested, the HPAB hydrogel showed the lowest degree of swelling (~170 g/g).

In Group 2, the incorporation of kaolinitic clay (HSAC) resulted in the lowest overall swelling capacity (~304 g/g). In contrast, hydrogels containing steatite (HPPS and HSPS) exhibited the highest swelling values, ranging from 280 to 340 g/g. In summary, the HSPS (polyacrylamide and steatite) formulation showed the best swelling performance in distilled water.

### 2.4. Swelling in Saline Solution

**Figure 4:**
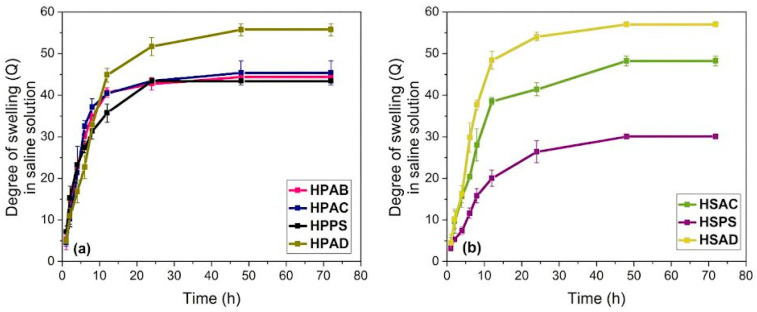
Swelling degree of the synthesized hydrogels in saline solution: (**a**) Group 1 (gum-based systems: HPAB, HPAC, HPPS, and HPAD) and (**b**) Group 2 (gum-free systems: HSAC, HSPS, and HSAD).

The hydrogels were evaluated for swelling in a 0.1 mol L^−1^ NaCl saline solution, as shown in Figure 4.

As shown in Figure 4, a significant reduction in the swelling capacity in aqueous saline solution (NaCl 0.1 mol L^−1^) was observed compared to that in distilled water. The hydrogels from Group 2 (without polysaccharide) showed a slight increase in swelling compared to Group 1. A remarkable behavior was observed for materials containing only soapstone (HPPS and HSPS), which exhibited the lowest swelling in saline solution. However, the systems containing kaolinitic clay and steatite (HPAD and HSAD) showed the greatest swelling in saline solution, reaching values of approximately 60 g/g.

**Figure 5:**
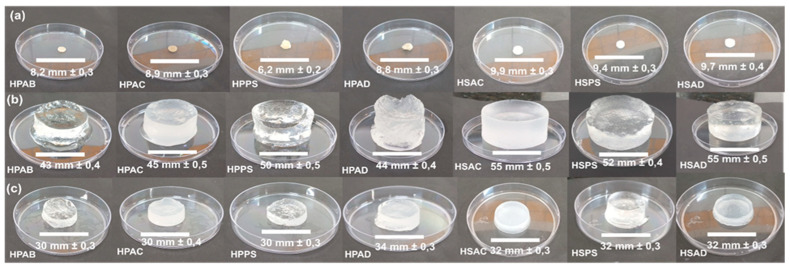
Visual appearance of the synthesized hydrogels: (**a**) dry state; (**b**) swollen in distilled water; and (**c**) swollen in aqueous saline solution (0.1 mol L^−1^ NaCl).

Figure 5 shows digital images of the hydrogels in the dry state and at swelling equilibrium state in distilled water and in aqueous saline solution (0.1 mol L^−1^ NaCl), allowing for a direct comparison of their macroscopic appearance across different formulations.

### 2.5. Swelling Kinetics of Hydrogels in Distilled Water and Aqueous Saline Solution

The dynamics of water absorption were evaluated in distilled water and aqueous saline solution (0.1 mol L^−1^ NaCl) to investigate the effect of ionic strength on the swelling rate. The experimental data were fitted to the pseudo-second-order model (Equations (1–3)) [26,27,28].

*t*/*Q* = *A* + *Bt* (1)

where

(2) $$A=\frac{1}{k_{s}Qt^{2}}$$

(3) $$B=\frac{1}{Qt}$$

**Figure 6:**
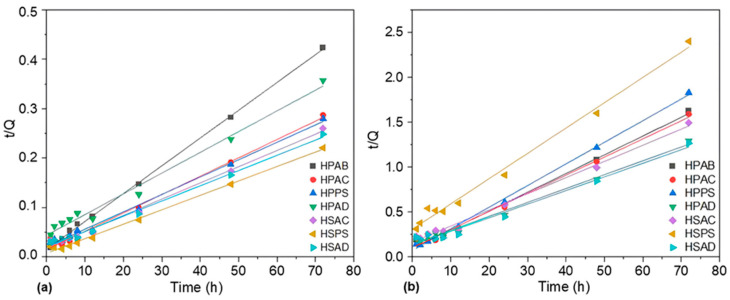
Linearized swelling kinetics curves for the synthesized hydrogels: (**a**) in distilled water and (**b**) in aqueous saline solution.

As shown in Figure 6, the model showed an excellent fit to the experimental data in both media (*R*^2^ > 0.97). In distilled water (Figure 6a), the smallest slopes and the greatest equilibrium capacities were observed. In contrast, the presence of Na^+^ ions in the saline medium (Figure 6b) resulted in a reduction in swelling. The kinetic parameters calculated for both media are detailed in Table 1 and Table 2.

In aqueous saline medium (Table 2), the kinetic behavior changed. The HPPS hydrogel showed rapid saturation (higher *k*_s_), but the lowest final swelling capacity. The HPAD hydrogel demonstrated slower swelling under salinity. In Group 2, HSAD followed a similar trend of slower swelling under saline conditions.

### 2.6. Hydrogel Swelling Sensitivity Factor in Saline Solution

The sensitivity of the hydrogels to ionic strength (0.1 mol L^−1^ NaCl) was quantified by the factor ƒ, which expresses the relative reduction in swelling in saline medium compared to distilled water, according to Equation (4) [27].

(4) $$ƒ=1-\left(\frac{Qsalt}{Qdistilled\;water}\right)$$

Higher ƒ values indicate that the polymer network is highly sensitive to the presence of electrolytes, whereas low values suggest greater structural stability against ionic shielding. The corresponding results are presented in Table 3.

According to Table 3, all hydrogels exhibited high ƒ values (0.73–0.90). The highest ƒ values were observed in formulations containing only soapstone (HSPS and HPPS). In contrast, hydrogels containing pure white angico gum (HPAB) and the hybrid composite formulation (HPAD) showed the lowest ƒ values.

### 2.7. Point of Zero Charge (PZC)

**Figure 7:**
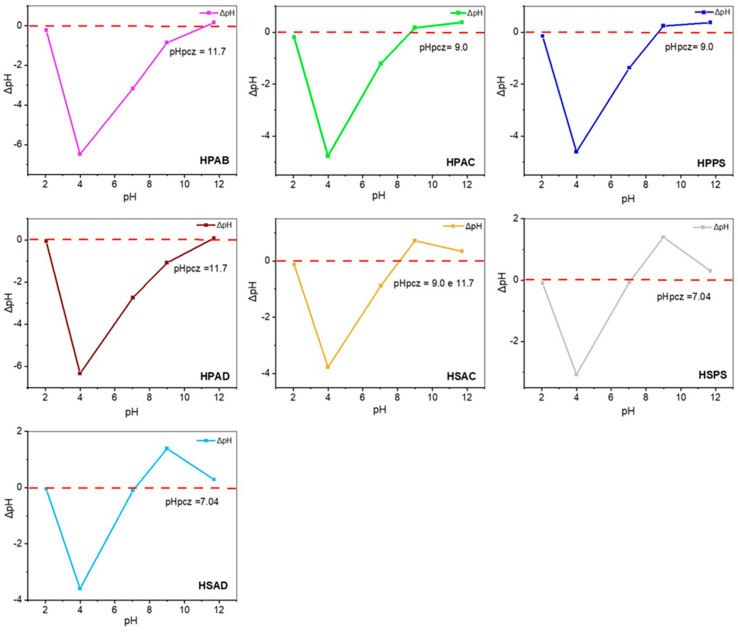
Point of zero charge (PZC) determination for the synthesized hydrogels (HPAB, HPAC, HPPS, and HPAD) and control hydrogels without white angico gum (HSAC, HSPS, and HSAD).

The electrostatic behavior of the hydrogel surfaces was evaluated by determining the PZC (Figure 7).

All hydrogels showed an inflection point at pH 4.0. The hydrogels from Group 1 (containing white angico gum) exhibited high PZC values ranging from pH 9.0 to 11.7. In Group 2 (without gum), most formulations showed PZC values close to neutrality (~7.0). Notably, the hydrogel containing only kaolinitic clay (HSAC) exhibited a PZC ranging from pH 9.0 to 11.7.

### 2.8. Scanning Electron Microscopy (SEM) Analysis

**Figure 8:**
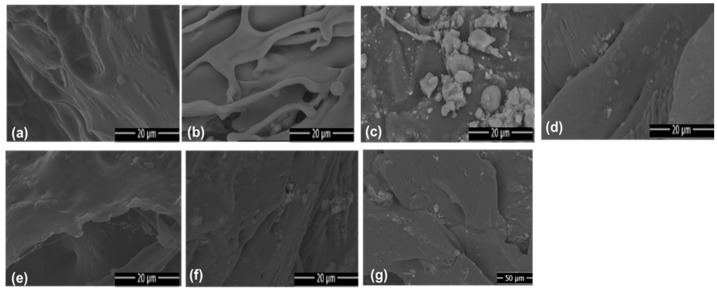
SEM micrographs of the synthesized hydrogel surfaces: (**a**) pure white angico gum hydrogel (HPAB); (**b**) gum/clay hydrogel (HPAC); (**c**) gum/soapstone hydrogel (HPPS); (**d**) gum/clay/soapstone hybrid hydrogel (HPAD); (**e**) clay/PAM hydrogel (HSAC); (**f**) soapstone/PAM hydrogel (HSPS); and (**g**) clay/soapstone/PAM hydrogel (HSAD).

Analysis of SEM micrographs allowed the determination of the surface morphology of the synthesized hydrogels (Figure 8). Hydrogels containing white angico gum exhibited a denser polymer network. In contrast, hydrogels without polysaccharides exhibited a more discontinuous network with cracks.

In formulations containing steatite (HPPS), attachment of cuboidal particles with a lamellar structure to the pore walls was observed (Figure 8c). For hydrogels containing kaolinitic clay (HPAC and HSAC), the morphology revealed agglomerates of small plate-like structures integrated into the matrix. Hydrogels combining both filler materials (HPAD and HSAD) exhibited a highly heterogeneous and porous morphology.

### 2.9. Thermogravimetric Analysis (TG)

**Figure 9:**
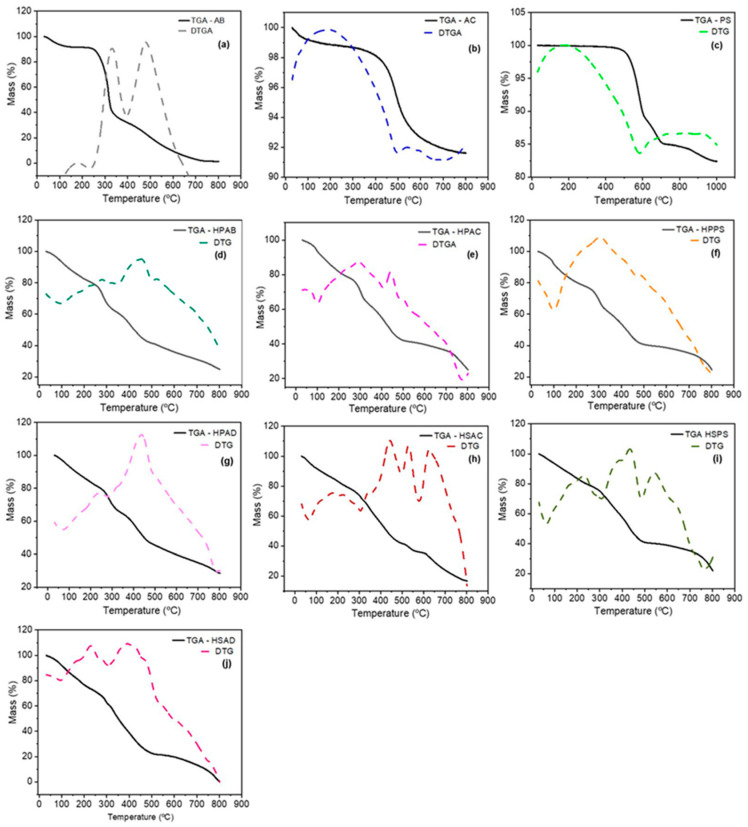
TG and DTG curves of (**a**) white angico gum (AB); (**b**) kaolinitic clay (AC); (**c**) soapstone (PS); (**d**–**g**) gum-based hydrogels (HPAB, HPAC, HPPS, and HPAD); and (**h**–**j**) gum-free hydrogels (HSAC, HSPS, and HSAD).

The thermal stability of the precursors and hydrogels was evaluated by TG and DTG (Figure 9).

Pure white angico gum (AB) exhibited its main thermal degradation event at 280 °C. After incorporation into the polymer matrix, this peak shifted to approximately 310 °C. Soapstone (PS) increased the maximum degradation temperature of the matrix from 403 °C to 412 °C and increased the inorganic residue content at 600 °C. The HPAD hydrogel exhibited the most stable thermal profile in the series. In contrast, the formulations without polysaccharides (Group 2) showed reduced initial mass loss.

### 2.10. Compression Test

**Figure 10:**
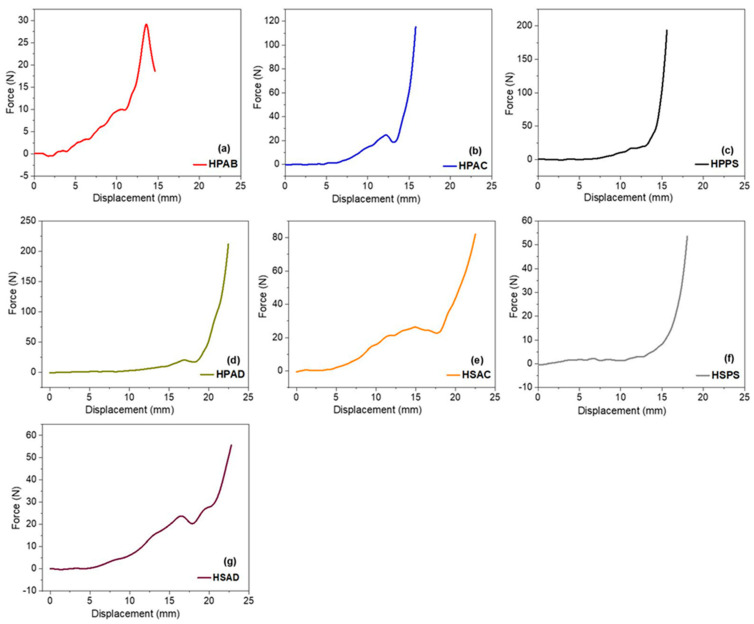
Force (N) versus displacement (mm) curves obtained from axial compression tests of the synthesized hydrogels.

The mechanical strength of the hydrogels was evaluated by means of axial compression tests (Figure 10), determining the maximum stress (*σ_max_*) and the elastic compression modulus (*E*).

The quantitative results are presented in Table 4. In Group 1, the hydrogel containing only white angico gum (HPAB) showed the lowest stiffness values (*σ_max_* = 0.0149 MPa; *E* = 0.013 MPa). In contrast, the incorporation of soapstone (HPPS) significantly increased the mechanical strength (*σ_max_* = 0.1615 MPa; *E* = 0.037 MPa). In Group 2, a similar trend was observed for HSPS, while the simultaneous combination of fillers in the absence of gum (HSAD) led to structural heterogeneities that reduced mechanical performance. The HPAD showed intermediate values compared to the others (*E* = 0.025 MPa).

### 2.11. Mass Loss Test of Hydrogels at 35 °C

**Figure 11:**
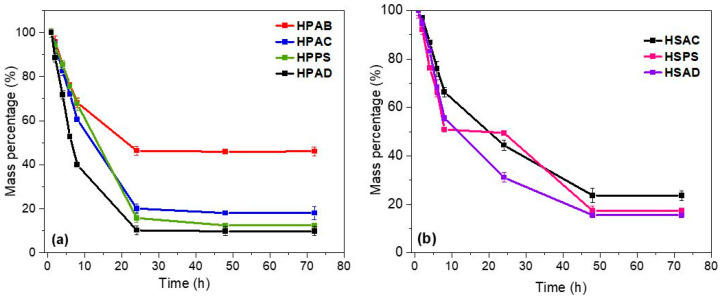
Mass loss profiles of the synthesized hydrogels after swelling in distilled water: (**a**) Group 1 (gum-based: HPAB, HPAC, HPPS, and HPAD) and (**b**) Group 2 (gum-free: HSAC, HSPS, and HSAD).

The water retention capacity of the hydrogels at 35 °C was evaluated through the evaporation mass loss profile (Figure 11).

All formulations exhibited a two-stage dehydration profile: an initial linear water loss (up to 10 h), followed by a slower stage. The hydrogels in Group 1 (with AB) reached dehydration equilibrium in approximately 25 h, while Group 2 (without AB) required about 50 h.

The HPAB (pure gum) and HSAC (kaolinitic clay only) hydrogels showed less pronounced dehydration slopes. In contrast, samples containing hybrid fillers (HPAD and HSAD) exhibited higher water loss rates.

### 2.12. Phosphate Release Test

**Figure 12:**
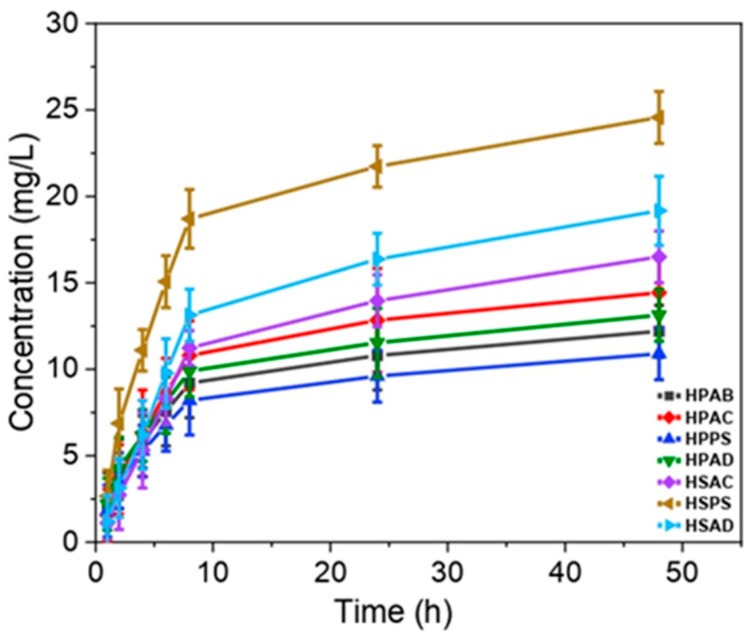
Phosphate release profiles of the synthesized hydrogels: gum-based (HPAB, HPAC, HPPS, and HPAD) and gum-free (HSAC, HSPS, and HSAD) systems. *Y*-axis: cumulative concentration (mg mL^−1^).

The phosphate release profile was evaluated at 35 °C (Figure 12) to assess the efficiency of the hydrogels as controlled nutrient delivery systems.

The HPAC hydrogel (polyacrylamide/gum/kaolinitic clay) showed the highest nutrient retention in Group 1. In contrast, HPPS (gum/soapstone) showed lower phosphate retention. HSPS (soapstone/without gum) showed the highest phosphate retention in its group. In contrast, HSAC (polyacrylamide/kaolinitic clay/without gum) showed the lowest performance in the group.

**Figure 13:**
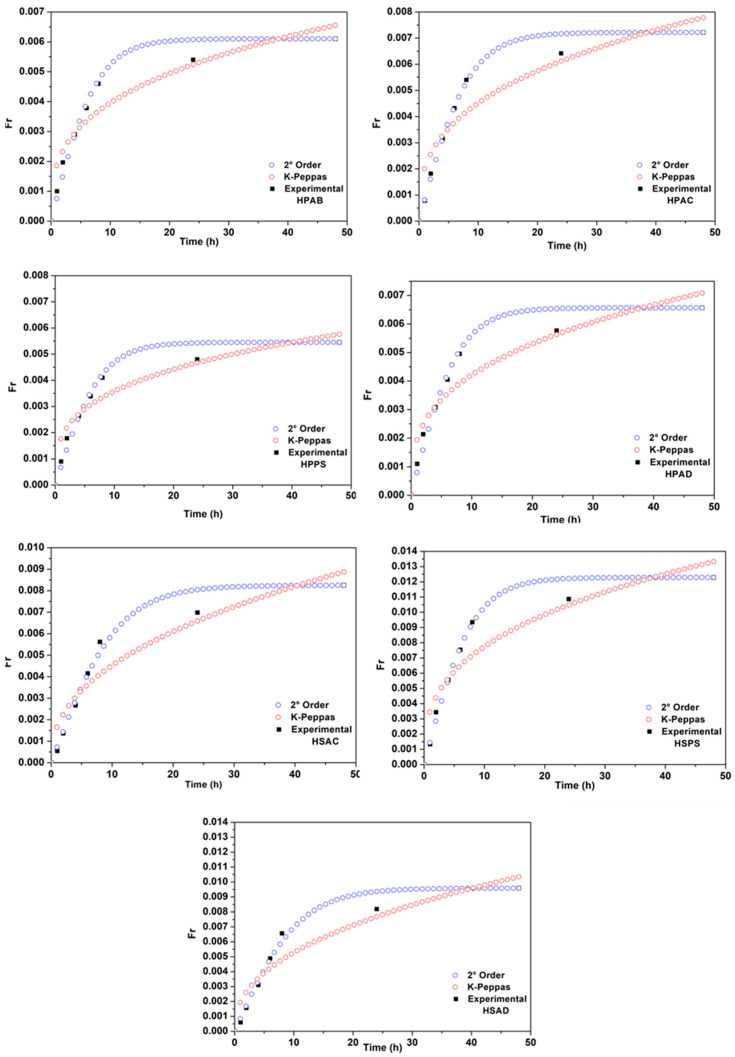
Kinetic curves for phosphate release profiles of the synthesized hydrogels: gum-based (HPAB, HPAC, HPPS, and HPAD) and gum-free (HSAC, HSPS, and HSAD), fitted to mathematical models: reversible second-order [29] and Korsmeyer–Peppas models [30].

To understand the transport mechanism, the data were fitted to the reversible second-order [29] and Korsmeyer–Peppas [30] models, as shown in Figure 13, and the model fitting data are presented in Table 5.

The reversible second-order model presented higher coefficients of determination (*R*^2^ > 0.98) for all compositions, surpassing the Korsmeyer–Peppas model.

### 2.13. Toxicity Test of Hydrogels with Artemia Salina

The survival parameters of *Artemia salina* nauplii for the different formulations are summarized in Table 6.

The results revealed that all formulations tested exhibited low toxicity, with survival rates exceeding 80%. The HPPS hydrogel (gum/soapstone) demonstrated the best ecotoxicity performance. In contrast, HPAC (gum/kaolinitic clay) showed slightly lower survival, although still within the safe range.

## 3. Discussion

The selection of the formulation containing 5% kaolin clay and soapstone was based on the superior performance of kaolin, as reported in literature reviews, as well as the better performance of this formulation in laboratory-synthesized hydrogels. According to several studies, such as those by Zhang [31], Brito [9], and Cheng [32], formulations containing between 5% and 10% kaolin provide higher water retention and greater absorption capacity in hydrogels compared to hydrogels without this component [9,32]. More promising results were obtained with the formulation containing 5% kaolin. The choice of the 5 wt% formulation represents a safer approach for swelling parameters (Q), since contents significantly above or below the optimal formulation can directly affect the swelling degree of these hydrogels. As the amount of kaolin increases, the Q value may gradually decrease [9,32]. A high kaolin content may favor the formation of additional crosslinking points within the hydrogel polymer network, increasing the composite’s crosslinking density, which reduces polymer elasticity and consequently results in a lower swelling degree [9,32]. A kaolin content lower than the optimal level may not produce the expected effects of incorporating the clay mineral, such as contributing to material hydrophilicity, as well as modulating the hydrogel’s mechanical properties [9]. Similarly, despite the limited number of studies on soapstone as a soil conditioner, the same 5% proportion was adopted for soapstone.

FTIR analysis confirmed the formation of the hybrid network and the preservation of the glycosidic structure of white angico gum, evidenced by bands at 1368 and 1029 cm^−1^ [24,25]. The broadening of the band in the 3500–3000 cm^−1^ region indicates overlapping of O–H and N–H stretching vibrations, confirming an increase in hydrogen bonding that ensures cohesion between the organic and inorganic phases [32,33,34]. The extent of hydrolysis was evidenced by the peaks at 1670 cm^−1^ and 1564 cm^−1^, which are essential for network expansion via electrostatic repulsion of carboxylate groups [9]. Additionally, the shift in the band at 3400 cm^−1^ confirms stable interactions with the mineral surfaces [9,23]. Unlike purely synthetic systems, the combination of gum with minerals creates a reinforcing network that attenuates the mechanical fragility typical of superabsorbent hydrogels. While carboxylates ensure absorption, minerals and polysaccharides provide structural stability. These results corroborate the synergistic interaction between the components, enabling the material to withstand real field pressures and conditions, and qualifying it as a potential resilient soil conditioner for agricultural management.

The broad X-ray diffraction halo of the gum is characteristic of the amorphous nature of natural polysaccharides, and the preservation of this profile in hydrogels indicates successful polymerization of acrylamide and the formation of a three-dimensional network [9]. The reduction in halo intensity suggests effective integration of the macromolecule into the polymeric matrix [35]. In composites, the attenuation or disappearance of mineral peaks indicates interactions between the polymer matrix and silicate layers, signaling good dispersion of the filler in the polyacrylamide network [36]. The persistence of residual kaolinite peaks reinforces that the lamellar structure of the mineral interacts with the functional groups of the matrix while preserving its crystalline identity [9]. The structural disorganization (amorphous nature) observed in all materials is a positive factor for the intended application, as amorphous structures facilitate polymer chain relaxation. This state promotes swelling and diffusion of water and nutrients, such as phosphate [35]. Thus, the XRD results corroborate the FTIR analyses, confirming the synthesis of hybrid materials with a microstructure favorable for water retention and the mechanical flexibility required for agricultural applications.

The reduction in swelling of Group 1 is attributed to the formation of a semi-interpenetrating (semi-IPN) network between the white angico gum and polyacrylamide; interactions between the chains increase the crosslinking density due to entanglements and elastic constraints, limiting volumetric expansion [37]. In the case of HSAC, the performance is due to the layered (lamellar) structure of kaolinite clay, whose surface hydroxyl groups act as physical crosslinking points through hydrogen bonding, increasing the rigidity of the network [38,39].

For hydrogels containing steatite, the behavior is explained by the presence of silanol groups (Si–OH) on the edges of the lamellae, which promote interactions with water despite the hydrophobic nature of talc [11]. Steatite acts as a spacer, weakening the strength of the polyacrylamide network and facilitating the penetration of water molecules into the matrix [11,19]. Overall, the results demonstrate that the selection of the mineral filler and the presence of the biopolymer allow for modulation of water retention, making the material versatile for soil water management.

The reduction in hydrogel absorption in saline solution is attributed to the charge shielding effect (electrostatic screening), in which cations interact with carboxylate groups, reducing chain-anionic repulsion and osmotic pressure [40]. The absence of white angico gum reduces steric hindrance, facilitating network expansion and conferring greater elasticity to the polyacrylamide chains [41,42]. In formulations containing steatite, the hydrophobic nature of talc accelerates network collapse under reduced swelling forces [10,11]. The success of the HPAD system is due to a synergistic effect: kaolinitic clay provides structural reinforcement against collapse [43], while steatite acts as a spacer, creating microporosity that facilitates solvent entrance and further diffusion [11]. This ability to maintain free volume under adverse conditions reinforces the potential of HPAD (gum/kaolinitic clay/steatite) as a resilient soil conditioner, ensuring durability during field hydration cycles.

According to swelling kinetics, the swelling behavior in distilled water is consistent with the Flory–Huggins theory for polymer–solvent mixtures [44]. In the case of HPPS, rapid attainment of equilibrium swelling is associated with the hydrophobic nature of talc; under conditions of ionic shielding, the network collapses rapidly due to the lack of hydrophilic surface groups that sustain hydration channels [16,45]. In HPAD, the delay in swelling results from the synergistic effect of the charges and branching of the white angico gum, which increases steric hindrance [39]. For HSAD (PAAm/kaolinitic clay/soapstone), the high mineral content creates physical barriers that require more time for osmotic pressure to overcome the mechanical resistance of the network [31,46]. From an agronomic point of view, this stable structure is desirable because it resists mechanical pressure from the soil, promotes prolonged moisture retention, and prevents rapid water loss [47].

Based on the analysis of the swelling sensitivity factor, Equation (4) (ƒ = 1 − (Q_salt_/(Q_distilled water_)) shows that, using the swelling capacity of the hydrogel in saline solution (Q_sal_) and in distilled water (Q_distilled water_), it is possible to evaluate the responsiveness of the hydrogel to ionic environments, such as soil conditions [27]. Values of ƒ close to 1 indicate a high dependence of the hydrogel swelling behavior on the ionic strength of the medium, i.e., greater environmental responsiveness [48]. For potential soil applications, lower values of ƒ indicate reduced sensitivity to ionic conditions and, therefore, higher stability of the hydrogel in saline (ionic) environments. The greater structural stability of HPAB and HPAD in saline media is attributed to the presence of the polysaccharide, whose hydroxyl groups favor hydrogen-bonding interactions, which are less affected by ionic strength variations than purely electrostatic interactions [32]. In the case of HPAD, the synergistic effect between the gum and kaolinitic clay promotes additional physical reinforcement, preserving network integrity even under ionic stress [49]. These results corroborate the equilibrium swelling and kinetic analyses, demonstrating that the incorporation of white angico gum and kaolinitic clay allows for modulation of the material’s response to salinity. For agricultural applications, this modulation is strategic because it ensures that the hydrogel maintains its functional water retention capacity even in soils with varying salt concentrations.

The inflection point at pH 4.0 marks the transition at which carboxylic groups become deprotonated, leading to electrostatic repulsion between anionic groups and network expansion [50,51]. In Group 1, the shift in the PZC suggests that the polysaccharide promotes electrostatic shielding of acidic groups and introduces new charge densities [30]. Under soil conditions (pH < PZC), this net positive charge facilitates the retention of anionic nutrients, such as phosphates [45]. In Group 2, the PZC close to neutrality reflects the equilibrium between amide and mineral surface groups (positive) and carboxylates (negative) [36]. The behavior of HSAC is justified by the strong protonation of the aluminol (Al–OH) groups of kaolinite, which masks the acidic sites of the matrix [43]. In summary, the composition allows the PZC to be modulated: Group 1 acts as a strategic anion retainer, while Group 2 functions as a versatile ion exchanger with tunable affinity for soil management [52].

As revealed by SEM analyses, the incorporation of white angico gum in Group 1 hydrogels resulted in materials with denser polymer walls and more pronounced surface roughness compared to Group 2 [53]. The fissures observed in Group 2 reflect greater structural fragility of the purely synthetic mineral matrix. In the case of soapstone, talc particles act as physical spacers due to the hydrophobic nature of their basal planes; this prevents excessive collapse of polymer chains during drying and preserves hydration channels, justifying the high swelling observed in these materials [11,53]. The clay plates in the HPAC and HSAC systems act as mechanical reinforcement of the network due to their affinity with the polar groups of polyacrylamide and gum [9]. The heterogeneous and porous morphology of hybrid systems (HPAD and HSAD) results from synergistic interactions between clay minerals and the polymeric phase [53]. This increased porosity is considered essential for solvent and nutrient transport, corroborating the effectiveness of hybrid fillers in optimizing hydrogel functionality in soil applications.

The increased thermal stability of the gum is attributed to steric confinement imposed by the polyacrylamide network in the semi-IPN system [54,55]. The mineral fillers act through a barrier effect: kaolinitic clay establishes hydrogen-bonding interactions that retard thermo-oxidation of the matrix [56], while soapstone acts as a thermal barrier that reduces heat transfer and restricts polymer mobility [57,58]. In HPAD, the combination of minerals maximizes internal tortuosity, increasing the apparent activation energy for thermal degradation [23]. Synergistic interactions with the polysaccharide occur through intermolecular interactions and interfacial reinforcement, contributing to the overall stability of the system. From an agricultural perspective, the thermal robustness of HPAD ensures structural integrity under high field temperatures. This supports a slower and more controlled degradation profile, preserving the resilience and water retention capacity of the hydrogel throughout the growth cycle.

The poor mechanical performance of HPAB suggests that the branched polysaccharide chains act as spacers, reducing the effective crosslinking density and increasing the viscoelasticity and flexibility of the network [51,59]. In contrast, the increased strength in HPPS indicates that soapstone acts as a lamellar filler that restricts polymer mobility and promotes stress redistribution [23,36]. Its layered morphology favors the sliding of structural planes, allowing for deformation without fracture [60]. In the case of HSAD, the loss of performance is attributed to heterogeneities generated by the mixture of fillers in the absence of the biopolymer, which normally acts as an interfacial compatibilizer [59]. The stability of HPAD confirms that the synergistic interaction between the gum and the minerals establishes strong interfacial cohesion. This cohesion restricts chain mobility, allowing the material to combine dimensional stability and biodegradability under soil water stress.

The mass loss tests of the hydrogels showed two stages of dehydration. The first stage corresponds to the evaporation of surface water, and the second is governed by the diffusion of water from the interior of the network to the surface [60,61]. The HPAB (pure gum) and HSAC (kaolinitic clay only) hydrogels exhibited higher water retention capacity compared to the other formulations, behaving as water-retaining matrices that maintain soil moisture for extended periods [62]. This behavior is attributed to strong hydrogen-bonding interactions between water and the functional groups of the gum, combined with the physical barrier effect imposed by kaolinitic clay, which hinders water vapor escape [32,63]. In contrast, samples containing simultaneous hybrid fillers (HPAD and HSAD) exhibited faster mass loss kinetics. Excess mineral content in the network can generate structural heterogeneities that create diffusion pathways for water vapor, accelerating dehydration [64,65].

The superior performance of the HPAC phosphate release assay is attributed to the synergy between the hydroxyl groups of the biopolymer and the surface charges of the clay. AC contributed to the formation of a denser hybrid network, enhancing phosphate retention through electrostatic interactions within a compact matrix [66,67]. The faster leaching of HSAC is attributed to its more porous morphology, observed by SEM, highlighting the critical role of the polysaccharide in maintaining system cohesion [36]. For soapstone-based materials, the release mechanism was predominantly physical. In HPPS, the interfacial incompatibility between hydrophobic talc and hydrophilic gum resulted in a more brittle structure, facilitating diffusion [11]. On the other hand, in HSPS, lamellar talc particles act as barriers that increase the tortuosity of the network and delay nutrient release [23].

The reversible second-order kinetic model describing phosphate release suggests that the process is governed by diffusion and ion exchange, in which the thermodynamic affinity between phosphate anions and active sites of the network (amide and hydroxyl groups) promotes nutrient retention in the solid phase [36,37].

According to the literature, survival rates above 80% indicate biocompatible and environmentally safe materials, without inhibitory effects on the ecosystem [30,68]. The superior performance of HPPS can be attributed to the mineral composition of steatite; talc and associated silicates may release trace micronutrients, such as calcium and potassium, which may contribute to biological activity, mitigating potential toxic effects [11,19]. The slightly lower survival rate of HPAC is associated with the physicochemical properties of kaolinitic clay, whose higher cation exchange capacity (CEC) and surface acidity can interact more strongly with the microenvironment, resulting in greater residual effects compared to the lower surface reactivity of soapstone [9,52]. In summary, the tests confirm that the incorporation of AB gum and mineral fillers does not compromise the biological viability of the systems. The demonstrated safety qualifies these hybrid hydrogels as sustainable inputs for agricultural management, minimizing the risk of environmental contamination.

## 4. Materials and Methods

### 4.1. Materials

The reagents acrylamide (AAm, 98% purity), potassium bicarbonate (KHCO_3_, 98% purity), N,N,N’,N’-tetramethylethylenediamine (TEMED, 98% purity), potassium persulfate (K_2_S_2_O_8_, 98% purity), and the crosslinking agent N,N’-methylenebisacrylamide (MBA, 98% purity) were purchased from Sigma-Aldrich (St. Louis, MO, USA). White angico gum (*Anadenanthera colubrina*) (AB) was obtained from natural exudates collected in the municipality of Rio Grande do Piauí (Piauí, Brazil). The mineral fillers consisted of kaolinitic clay (AC), from Oeiras (Piauí, Brazil), and steatite powder (PS), supplied by the São José mining company (Ouro Preto, Minas Gerais, Brazil). The particle size distribution of the mineral fillers was determined by mechanical sieving using mesh sizes of 30, 100, and 250. The analysis was performed in triplicate. Kaolinitic clay exhibited a predominant particle size distribution between 63 and 150 µm, while steatite was mainly composed of particles smaller than 63 µm. All reagents were used without further purification, except for the natural gum.

### 4.2. Methods

#### 4.2.1. Isolation and Purification of White Angico Gum

The purification followed a methodology adapted from Silva et al. [12]. Approximately 30 g of crude gum were dissolved in 150 mL of distilled water under stirring with hydrogen peroxide (H_2_O_2_, 90% purity). After the addition of excess sodium chloride (NaCl, 90% purity) (0.5 g 100 mL^−1^) and adjustment of the pH to 7.0 with NaOH (0.1 mol L^−1^, 95% purity), the solution was further filtered. The polysaccharide was precipitated in 95% ethanol; washed successively with ethanol, water and acetone; dried in an oven at 50 °C; and macerated until a fine powder was obtained.

#### 4.2.2. Synthesis and Hydrolysis of Hydrogels

Hydrogels were synthesized via radical polymerization in aqueous solution. For formulations containing the polysaccharide, 2 g of purified gum were dissolved in 30 mL of distilled water. Then, 2 g of AAm, 0.024 g of MBA, 0.016 g of K_2_S_2_O_8_, 0.1 g of KHCO_3_ and 0.2 mL of TEMED were added. The system was kept under stirring (150 rpm) and a nitrogen atmosphere for 30 min for gelation [9]. The samples were divided into two experimental groups based on the mass of additives relative to the monomer (AAm). Each hydrogel contains approximately 2 g of acrylamide in its formulation. The 1:1 ratio is approximately 2 g of acrylamide to 2 g of polysaccharide, in the case of the hydrogels in Group 1. The 5% proportion of kaolin or steatite is based on the mass content relative to the acrylamide monomer. Group 1, hydrogels with polysaccharide: HPAB, purified white angico gum (1:1); HPAC, white angico gum (1:1) with 5% (w/w) kaolin (kaolinitic clay); HPPS, white angico gum (1:1) with 5% (w/w) steatite (soapstone); HPAD, white angico gum (1:1) with 5% (w/w) kaolin and 5% (w/w) steatite (soapstone); Group 2, hydrogels without gum, with polyacrylamide: HSAC, 5% (w/w) kaolin; HSPS, 5% (w/w) soapstone; HSAD, 5% (w/w) kaolin and 5% (m/m) steatite. After synthesis, all materials were subjected to alkaline hydrolysis in a NaOH solution (0.1 mol L^−1^) at a ratio of 1 g of polymer to 40 mL of solution, heated to 50 °C for 1 h. This process aimed to convert amide groups into carboxylates to enhance the water absorption capacity. Finally, the hydrogels were washed with distilled water and lyophilized for subsequent characterizations.

The synthesized hydrogels were characterized using several analytical techniques. Structural properties and functional groups were analyzed by Fourier transform infrared spectroscopy (FTIR) using a Perkin Elmer Spectrum 100 spectrometer (Waltham, Massachusetts, USA), while the crystalline structure of the precursors and hydrogels was evaluated by X-ray diffraction (XRD) (Shimadzu, Japan). Surface morphology and porosity were examined via scanning electron microscopy (SEM) (FEI Company, Hillsboro, Oregon, EUA). Thermal stability and degradation profiles were determined through thermogravimetric analysis (TG/DTG) (Shimadzu Corporation, Kyoto, Japan), and mechanical performance (Shimadzu Corporation, Kyoto, Japan), including maximum compressive strength and elastic modulus, was assessed using compressive tests.

Furthermore, the functional performance was evaluated through standardized calculations. The swelling degree (*Q*) was measured in both distilled water and saline solution (0.10 mol L^−1^ NaCl), and the salt sensitivity factor (*f*) and swelling kinetics were determined to understand the network’s water absorption capacity. Surface charge behavior was assessed by the point of zero charge (PZC). For agricultural application, phosphate release was monitored, and the release mechanisms were investigated using Korsmeyer–Peppas and reversible second-order mathematical models, with the fit quality verified by the coefficient of determination (*R*^2^). Finally, environmental safety was evaluated through acute toxicity assays using *Artemia salina* nauplii to determine survival percentages across different concentrations.

## 5. Conclusions

Based on a comparative analysis of all synthesized hydrogel formulations, it was observed that compositions containing approximately 5–10% kaolinitic clay (AC) exhibited better performance in the laboratory tests conducted. In this context, the composition containing 5% kaolinitic clay and 5% steatite (PS) was used as a reference for the development of the different formulations evaluated in this study, with and without polysaccharide incorporation. Among all tested formulations, the HPAD hydrogel, which combines polyacrylamide, white angico gum, kaolinitic clay, and steatite, showed the best overall performance. This formulation exhibited the highest compressive strength (>200 N), improved thermal stability (up to 310 °C), and better swelling capacity in saline medium (approximately 60 g/g) compared to the other samples.

This study confirmed the successful synthesis of semi-IPN hybrid hydrogels based on polyacrylamide, white angico gum, and mineral fillers. FTIR, XRD, and SEM analyses demonstrated the synergistic integration of the components, resulting in a highly porous architecture with thermally stable characteristics. The interconnected pore network contributes to water retention even under desiccation conditions. The enhanced swelling capacity observed is closely related to this porous morphology.

Functional tests showed that the incorporation of PS and AC enables modulation of the material’s properties: while PS promotes higher swelling (>300 g/g) and contributes to mechanical reinforcement (E = 0.035–0.037 MPa), AC and white angico gum favor improved water retention and sustained phosphate release governed by diffusion and adsorption/desorption processes, driven by the affinity between phosphate anions and active sites (amide and hydroxyl groups). Adjustment of the point of zero charge (PZC) between pH 7.0 and 11.7 further confers versatility as ion exchangers for tropical soils.

All tested hydrogels exhibited low acute toxicity, with Artemia salina survival rates exceeding 80%, confirming their biocompatibility and environmental safety. These findings consolidate the developed hydrogels as potential sustainable inputs for agricultural management, minimizing the risk of environmental contamination.

Looking ahead, controlling the loading density and tortuosity of the polymeric matrix offers a promising pathway for the potential development of smart fertilization systems and resilient soil conditioners capable of optimizing water retention and nutrient delivery, thereby enhancing agricultural productivity under water-scarce conditions.

## Figures and Tables

**Table 1:** Swelling kinetics parameters obtained from the pseudo-second-order model for the synthesized hydrogels in distilled water.

Hydrogel	*Q* Experimental (g/g)	*Q* Predicted (g/g)	Time to Reach Equilibrium (h)	*R*^2^	*k*_s_ (*h*^−1^)
HPAB	177.94	168.13	48	0.99945	2.010 × 10^−3^
HPAC	269.54	249.04	48	0.98985	0.939 × 10^−3^
HPPS	284.09	254.00	50	0.99472	0.595 × 10^−3^
HPAD	238.66	197.38	50	0.98674	0.400 × 10^−3^
HSAC	304.88	273.24	48	0.99431	0.589 × 10^−3^
HSPS	342.47	324.46	48	0.99518	1.090 × 10^−3^
HSAD	324.68	284.83	48	0.99400	0.456 × 10^−3^

**Table 2:** Swelling kinetic parameters of the synthesized hydrogels in saline solution, obtained from the pseudo-second-order model.

Hydrogel	*Q* Experimental (g/g)	*Q* Predicted (g/g)	Time to Reach Equilibrium (h)	*R*^2^	*k*_s_ (h^−1^)
HPAB	48.01	43.87	48	0.99372	4.62 × 10^−3^
HPAC	49.63	44.81	48	0.98961	3.90 × 10^−3^
HPPS	41.60	39.22	50	0.99816	7.94 × 10^−3^
HPAD	64.85	54.66	50	0.98910	1.66 × 10^−3^
HSAC	55.65	46.77	48	0.99305	1.97 × 10^−3^
HSPS	35.55	28.93	48	0.99158	2.56 × 10^−3^
HSAD	66.09	55.86	48	0.98208	1.71 × 10^−3^

**Table 3:** Sensitivity factor (ƒ) of the synthesized hybrid hydrogels.

Sample	*Q* Distilled Water (g/g)	*Q* Salt (g/g)	*f* (Sensitivity to Salt)
HPAB	177.94	48.01	0.730
HPAC	269.54	49.63	0.816
HPPS	284.09	41.60	0.854
HPAD	238.66	64.85	0.728
HSAC	304.88	55.65	0.818
HSPS	342.47	35.55	0.896
HSAD	324.68	66.09	0.796

**Table 4:** Maximum compressive stress (*σ_max_*) and elastic modulus (E) of the synthesized hydrogels.

Hydrogel	Maximum Compression Stress (*σ_max_*, MPa)	Compression Elastic Module (*E*, MPa)
HPAB	0.0149	0.013
HPAC	0.0499	0.022
HPPS	0.1615	0.037
HPAD	0.0951	0.025
HSAC	0.0249	0.016
HSPS	0.0771	0.035
HSAD	0.0234	0.016

**Table 5:** Coefficients of determination (*R*^2^) for the phosphate release kinetics of the synthesized hydrogels.

Hydrogels	*R*^2^ (2nd Order)	*R*^2^ (Korsmeyer–Peppas)
HPAB	0.987	0.933
HPAC	0.991	0.915
HPPS	0.986	0.935
HPAD	0.986	0.934
HSAC	0.982	0.921
HSPS	0.989	0.917
HSAD	0.982	0.917

**Table 6:** Survival percentages of Artemia salina nauplii at different hydrogel concentrations.

Time	Concentration (mg/mL)	HPAB	HPAC	HPPS	HPAD	HSAC	HSPS	HSAD
24h	5	0%	80%	0%	100%	100%	100%	90%
	1	100%	100%	100%	100%	100%	100%	100%
	0.5	100%	100%	100%	100%	100%	100%	100%
48h	5	0%	50%	0%	90%	90%	90%	80%
	1	90%	80%	100%	100%	90%	100%	90%
	0.5	100%	90%	100%	100%	90%	100%	90%

## Data Availability

The raw data supporting the conclusions of this article will be made available by the authors on request.
