# Correction: Carvalho et al. Novel Superabsorbent Hydrogels Based on Polyacrylamide and White Angico Gum Enhanced with Kaolinitic Clay and Soapstone for Potential Agricultural Applications. *Int. J. Mol. Sci.* 2026, *27*, 4150

**DOI:** 10.3390/ijms27125242

**Published:** 2026-06-10

**Authors:** Angelina Santos de Carvalho, Arthur Francisco de Paiva Alcântara, Vicente de Sousa Marques, Ariane Maria da Silva Santos, Ronaldo Cunha Coelho, Edvani Curti Muniz

**Affiliations:** 1 Graduate Program in Materials Science and Engineering, Federal University of Piauí, Teresina 64049-550, Brazil; angelinasantos653@gmail.com; 2 Teaching Department, Federal Institute of Piauí (IFPI), Valença Campus, Valença 64300-000, Brazil; arthur.alcantara@ifpi.edu.br; 3 Teaching Department, Federal Institute of Maranhão (IFMA), Codó Campus, Maranhão 65400-000, Brazil; vsmarques7@gmail.com; 4 Department of Chemistry, Federal University of Piauí, Teresina 64049-550, Brazil; ariane.am42@gmail.com; 5 Teaching Department, Federal Institute of Piauí (IFPI), Campus Teresina Central, Teresina 64000-040, Brazil; ronald@ifpi.edu.br; 6 Graduate Program in Chemistry, State University of Maringá, Maringá 87020-900, Brazil

## Error in Table

In the original publication [1], there was a mistake in Table 4 as published. The error lies in the values in Table 4. The corrected Table 4 appears below. The original publication has also been updated.

## Text Correction

There was an error in the original publication. The values for the elastic compression modulus had not been updated to the correct values, which is due to an error in Table 4.

A correction has been made to Section 5 (Conclusions), Paragraph Number 3.

Functional tests showed that the incorporation of PS and AC enables modulation of the material’s properties: while PS promotes higher swelling (>300 g/g) and contributes to mechanical reinforcement (E = 0.035–0.037 MPa), AC and white angico gum favor improved water retention and sustained phosphate release governed by diffusion and adsorption/desorption processes, driven by the affinity between phosphate anions and active sites (amide and hydroxyl groups). Adjustment of the point of zero charge (PZC) between pH 7.0 and 11.7 further confers versatility as ion exchangers for tropical soils.

The authors state that the scientific conclusions are unaffected. This correction was approved by the Academic Editor. The original publication has also been updated.

## Figures and Tables

**Table 4:** Maximum compressive stress (*σ_max_*) and elastic modulus (E) of the synthesized hydrogels.

Hydrogel	Maximum Compression Stress (*σ_max_*, MPa)	Compression Elastic Module (*E*, MPa)
HPAB	0.0149	0.013
HPAC	0.0499	0.022
HPPS	0.1615	0.037
HPAD	0.0951	0.025
HSAC	0.0249	0.016
HSPS	0.0771	0.035
HSAD	0.0234	0.016
